# Treatment of rosacea with upadacitinib and abrocitinib: case report and review of evidence for Janus kinase inhibition in rosacea

**DOI:** 10.3389/fimmu.2024.1416004

**Published:** 2024-07-09

**Authors:** Ting Zhang, Xu Liu, Lu Zhang, Xian Jiang

**Affiliations:** ^1^ Department of Dermatology, West China Hospital, Sichuan University, Chengdu, China; ^2^ Laboratory of Dermatology, Clinical Institute of Inflammation and Immunology, Frontiers Science Center for Disease-Related Molecular Network, West China Hospital, Sichuan University, Chengdu, China

**Keywords:** rosacea, JAK inhibitor, upadacitinib, abrocitinib, case report

## Abstract

**Introduction:**

Conventional rosacea treatments are not uniformly pervasive, and the adverse reactions can potentially constrain their utility. The clinical use of JAK1 inhibitors upadacitinib and abrocitinib in the treatment of refractory rosacea has rarely been explored.

**Case report:**

We presented two cases of patients who received the JAK1 inhibitor upadacitinib and four cases of patients who received the JAK1 inhibitor abrocitinib for the treatment of refractory rosacea.

**Discussion:**

The JAK1 inhibitors upadacitinib and abrocitinib may be promising medical options for patients with refractory rosacea. However, the long-term safety and efficacy of upadacitinib and abrocitinib require prospective controlled studies to assess them more comprehensively.

## Introduction

Rosacea, a prevalent chronic inflammatory skin disease, is characterized by frequent facial flushing, erythema, papules, and telangiectasia (1). It affects individuals of all races, predominantly women between the ages of 30 and 40 years. The global prevalence of rosacea is estimated to be 5.46%, with a slightly lower prevalence in the Chinese population at 3.48% (2, 3). At present, the exact underlying etiology of rosacea remains unclear. It may encompass hereditary components, innate immune system disorders, facial vascular regulatory dysfunction, neurogenic inflammation, skin barrier disruption, and elevated levels of Demodex mites (4–6). Traditional treatments include oral antibiotics, topical medications, and laser therapy, but are limited in effectiveness and prone to recurrence (7). Therefore, there is an urgent need for further research into additional therapeutic options for rosacea.

The Janus kinase-signal transducer and activator of transcription (JAK-STAT) signaling pathway has garnered significant interest due to its pivotal role in modulating immune and inflammatory responses (8). JAKs comprise a family of intracellular protein tyrosine kinases that transduce signals triggered by cytokines and growth factors to activate downstream STAT proteins, thereby affecting gene expression (9). Considering the central role of this pathway in the regulation of immune responses and inflammatory processes, strategies to inhibit this pathway have emerged as a new direction for the treatment of rosacea. JAK inhibitors demonstrate potent suppression of inflammatory signaling pathways driven by the JAK pathway, and are capable of modulating an array of cytokines, including interleukins (ILs) and interferons (10). Notably, selective JAK1 inhibitors have found widespread application in alleviating inflammatory conditions such as atopic dermatitis, rheumatoid arthritis, and psoriatic arthritis (11–13). Here, we report two cases of refractory rosacea treated with upadacitinib and three cases of refractory rosacea treated with abrocitinib.

## Materials and methods

### Patients

#### Case 1

A 28-year-old woman presented to West China Hospital with symptoms of facial erythema, dryness, flushing, burning, and pruritus. Following a thorough evaluation, she received a diagnosis of erythematotelangiectatic rosacea. Over the subsequent 2 years, she embarked on an extensive therapeutic regimen aimed at managing her condition.

#### Case 2

A 45-year-old woman presented to West China Hospital with facial erythema, dryness, tingling, and flushing. After a comprehensive assessment, she was diagnosed with erythematotelangiectatic rosacea and has since pursued a broad-based therapeutic approach spanning 3 years in an effort to control her symptoms.

#### Case 3

A 37-year-old woman presented to West China Hospital with complaints of facial erythema, dryness, burning, pruritus, and visible telangiectasias. She was diagnosed with erythematotelangiectatic rosacea and has undergone a comprehensive therapeutic journey over the past 4 years.

#### Case 4

A 41-year-old woman presented to West China Hospital with symptoms of facial erythema, dryness, and tingling. She was diagnosed with erythematotelangiectatic rosacea and has since engaged in an extensive therapeutic endeavor for 3 years to manage her disease.

#### Case 5

A 38-year-old woman came to West China Hospital complaining of facial erythema, burning, and pruritus. She was diagnosed with erythematotelangiectatic rosacea following a thorough evaluation. Over the next 3 years, she undertook a comprehensive therapeutic regimen aimed at alleviating her symptoms.

#### Case 6

A 35-year-old woman presented to West China Hospital with facial erythema, dryness, and flushing. She was diagnosed with erythematotelangiectatic rosacea and initiated an extensive therapeutic journey over the past 3 years.

### Diagnosis

The diagnosis of rosacea in this study adhered to the National Rosacea Society Expert Committee’s (NRSEC) diagnostic criteria for rosacea (Version 2017) (14). The inclusion criteria were as follows: (1) confirmed diagnosis of rosacea; (2) conventional treatments proven ineffective; (3) exclusion of differential diagnosis of lupus erythematosus, seborrheic dermatitis, etc; and (4) absence of cardiovascular disease and autoimmune disease.

### Examination and evaluation

All six patients in this report underwent a VISIA examination. Additionally, all patients provided blood samples for routine tests, including full and differential blood counts, liver function tests, renal function tests, electrolyte panels, lipid profiles, glucose levels, and viral screening for hepatitis B, hepatitis C, tuberculosis, and HIV. All laboratory results were within normal ranges for every patient. The severity of rosacea was evaluated according to the Clinician’s Erythema Assessment (CEA) (15) and the Investigator’s Global Assessment (IGA) (16). Regarding the severity rating of rosacea symptoms, a score of 0 indicates an absence of symptoms, 1 indicates nearly imperceptible symptoms, 2 indicates mild, 3 indicates moderate, and 4 indicates severe. The Hospital Anxiety and Depression Scale (HADS) was utilized to assess the psychological well-being of rosacea patients (17). To measure the impact on quality of life, both the rosacea-specific quality-of-life instrument (RosQol) and the Dermatology Life Quality Index (DLQI) were utilized (18, 19).

### Treatment

All six patients received comprehensive therapies and interventions that encompassed oral minocycline, hydroxychloroquine, carvedilol, antihistamines, antianxiety medications, vitamins, pregabalin, alprazolam, and sodium bromide, as well as physical therapy such as phototherapy and sonophoresis therapy. However, these interventions failed to alleviate these rosacea patients’ symptoms. Therefore, after careful evaluations, we prescribed upadacitinib (15 mg once daily) for cases 1 and 2, and abrocitinib (100 mg once daily) for cases 3–6.

### Follow-up and efficacy evaluation

These patients are followed up once a month and adhere to regular medication for at least 2 months. Subsequently, all patients continue to follow-up and decide whether to reduce or gradually stop the dosage based on the changes in the patient’s symptoms, willingness to be treated, and the presence of adverse effects to ensure patient safety and satisfaction. VISIA examination were performed, and therapeutic effects were evaluated at each follow-up.

## Results

### Case 1

**Figure 1:**
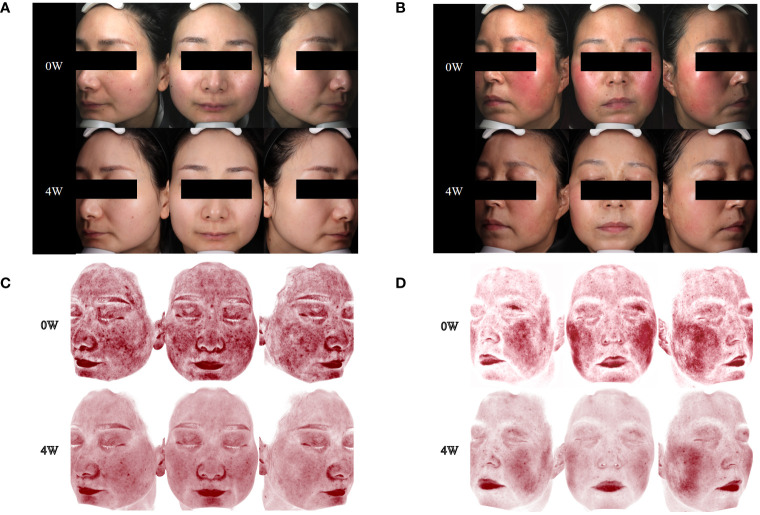
Changes in skin lesions and skin vascularity during upadacitinib treatment. **(A)** Case 1, skin lesions at 0 and 4 weeks of upadacitinib treatment. **(B)** Case 2, skin lesions at 0 and 4 weeks of upadacitinib treatment. **(C)** Case 1, skin vascularity at 0 and 4 weeks of upadacitinib treatment. **(D)** Case 2, skin vascularity at 0 and 4 weeks of upadacitinib treatment.

**Figure 2:**
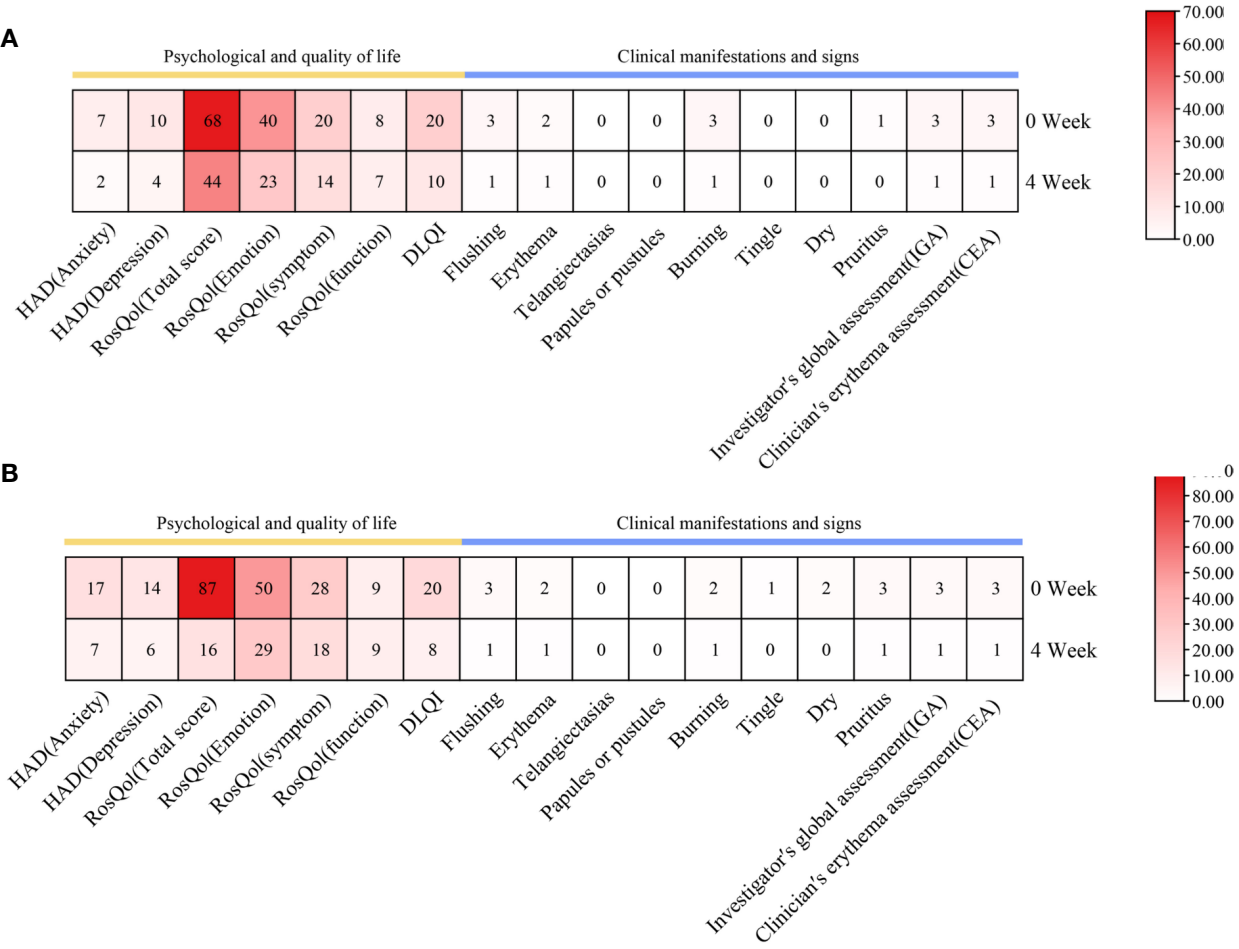
Changes in psychological well-being and quality of life, as well as clinical manifestations and signs, during upadacitinib treatment. **(A)** Case 1. **(B)** Case 2.

Within a mere 48-h interval following the initiation of upadacitinib treatment, this patient reported noteworthy improvement in symptoms such as erythema, flushing, and pruritus. At a follow-up visit only 2 weeks later, her facial erythema dramatically reduced, and her flushing, pruritus, and burning sensations were significantly improved. After 8 weeks of treatment, we reduced the dosage of upadacitinib to 15 mg every 2 days, and the patient experienced a transient rebound of erythema, flushing, and pruritus symptoms. After 12 weeks of treatment, we reduced the dosage of upadacitinib to 15 mg every 3 days, and the patient did not experience any exacerbation. During the therapeutic period, the patient’s facial erythema disappeared significantly (**Figures 1A, C**). Moreover, notable enhancements were noted in the patient’s psychological well-being and overall quality of life, paralleled by the gradual abatement of symptoms including erythema, flushing, and pruritus (**Figure 2A**).

### Case 2

During the 4 weeks of treatment with upadacitinib, this patient reported significant improvement in facial erythema, tingling, flushing, pruritus, and dry symptoms (**Figures 1B, D**). Moreover, this patient reported noteworthy improvements in changes in psychological well-being and quality-of-life (**Figure 2B**). Later, this patient was lost to follow-up due to personal reasons.

### Case 3

**Figure 3:**
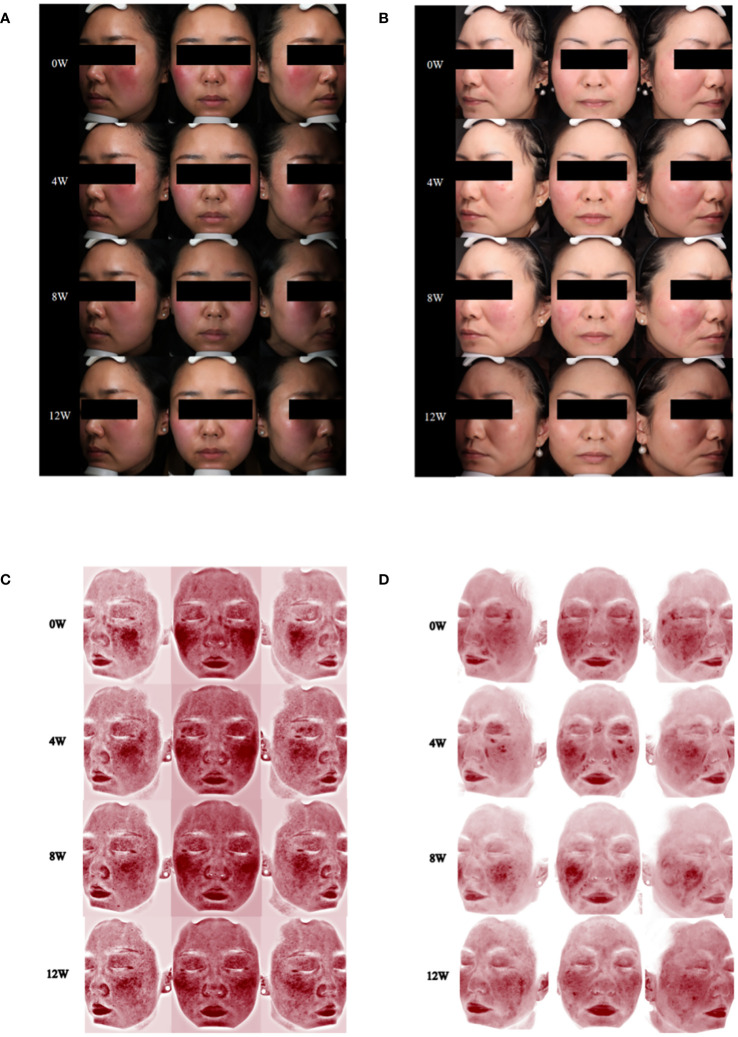
Changes of skin lesions and skin vascularity during abrocitinib treatment. **(A)** Case 3, skin lesions at 0, 4, 8, and 12 weeks of abrocitinib treatment. **(B)** Case 4, skin lesions at 0, 4, 8, and 12 weeks of abrocitinib treatment. **(C)** Case 3, skin vascularity at 0, 4, 8, and 12 weeks of abrocitinib treatment. **(D)** Case 4, skin vascularity at 0, 4, 8, and 12 weeks of abrocitinib treatment.

**Figure 4:**
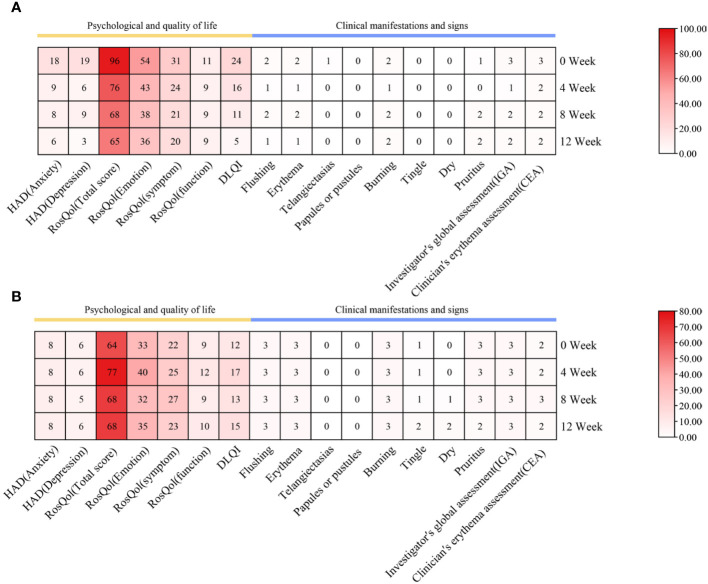
Changes in psychological well-being and quality of life, as well as clinical manifestations and signs during abrocitinib treatment. **(A)** Case 3. **(B)** Case 4.

Within a week following the initiation of abrocitinib treatment, this patient reported noteworthy improvement in burning and tingling symptoms. After 4 weeks of treatment with abrocitinib, this patient reported improvement in pruritus and life quality (**Figures 3A, 3C**, **4A**). After 16 weeks of treatment, we started to reduce the dosage of abrocitinib. Currently, the patient is receiving maintenance treatment with 100 mg of abrocitinib once every 4 days.

### Case 4

During the 20-week treatment of abrocitinib, this patient only showed mild improvement in pruritus and no significant improvement in quality of life (**Figures 3B, 3D**, **4B**). In the 20th week, the patient discontinued the medication due to hepatitis B DNA positivity and elevated transaminases on a retest.

### Case 5

During the 16 weeks of treatment with abrocitinib, this patient only showed mild improvement in pruritus, with no significant improvement in burning and tingle symptoms and no significant improvement in quality of life (**Supplementary Figure 1A**). Later, this patient was lost to follow-up due to personal reasons.

### Case 6

During the 12 weeks of treatment with abrocitinib, this patient showed no significant improvement in symptoms or quality of life (**Supplementary Figure 1B**). Currently, the patient has stopped using abrocitinib.

## Discussion

Rosacea is a chronic inflammatory skin disease with a complex pathogenesis involving multiple signaling pathways. Dysregulation of the innate immune system is involved in the pathogenesis of rosacea. Increased expression levels of cathelicidin, kallikrein-related peptidase 5 (KLK5), toll-like receptor 2 (TLR-2), and matrix metalloproteinase (MMP) have been observed in patients with rosacea (20–23). Notably, MMP-9 and TLR-2 can enhance KLK5, facilitating the conversion of cathelicidin into its active form, LL37, which promotes inflammation and angiogenesis (20). In addition, inflammatory cell infiltrates, including macrophages, mast cells, neutrophils, T cells, and B cells, have been identified in rosacea lesions. An imbalance in the CD4+/CD8+ T-cell ratio and Th1/Th17 cell polarization, along with elevated levels of associated cytokines such as interferon gamma (IFN-γ), tumor necrosis factor α (TNF-α), IL-6, IL-17, IL-22, STAT1, STAT3, STAT4, and vascular endothelial growth factor (VEGF), have been reported (24, 25). In terms of neurovascularity, it has been reported that VEGF expression is elevated in rosacea lesions, and VEGF can regulate angiogenesis and vascular permeability. In addition, various neuropeptides, such as pituitary adenylate cyclase-activating polypeptide (PACAP), vasoactive intestinal peptide, calcitonin gene-related peptide (CGRP), and substance P, are expressed at elevated levels in rosacea patients (26). These neuropeptides may play roles in the regulation of pain, inflammation, and vasodilation in rosacea patients.

The JAK/STAT pathway plays a pivotal role in the pathogenesis of rosacea. The JAK/STAT pathway intricately participates in the orchestration of inflammatory responses, and this signaling pathway upregulates the expression of proinflammatory cytokines, encompassing TNF-α, ILs such as IL-6, IL-8, and monocyte chemoattractant protein-1 (MCP1), which can cause facial flushing, erythema, and pruritus (27–31). Moreover, rosacea triggered by Demodex mite infestation may exacerbate inflammation through the activation of the JAK/STAT signaling pathway (32). Notably, a study identified STAT3 as a key gene linked to rosacea and skin barrier dysfunction using weighted gene coexpression network analysis (WGCNA). *In vivo* experiments further revealed that skin barrier dysfunction significantly elevates STAT3 expression and augments CD4+ T-cell infiltration in LL37-induced rosacea-like lesions in mice (33).

Based on rosacea pathology, we propose several mechanisms through which the JAK/STAT pathway may contribute to its development. In terms of innate immunity, TLR-2 activation can trigger the activation of the JAK/STAT3 pathway (34). The activated pathway can generate MMP-9 by releasing proMMP-9, which subsequently cleaves KLK5 into its active form. This process facilitates the conversion of cathelicidin into LL37, leading to erythema, telangiectasia, and inflammation (20, 22, 23). As for acquired immunity, patients with rosacea are reported to exhibit elevated TNF-α, IFN-γ, and IL-6, which can activate the JAK/STAT pathway (24, 35). The activation of this signaling pathway can regulate cytokines such as VEGF, influencing vascular permeability and angiogenesis. This, in turn, causes symptoms like flushing, erythema, and telangiectasia and promotes fibrosis (36, 37). In addition, the activation of STAT1/3 can promote the production and release of reactive oxygen species (ROS), which can mediate the inflammatory response via TLR2 receptors and induce vasodilation via neurogenic receptors (38–41). As previously mentioned, studies have also found that neurotransmitters such as vasoactive intestinal peptide, PACAP, and substance P are elevated in rosacea patients (26). On the other hand, substance P can induce phosphorylation of JAK2, STAT3, and STAT5, while vasoactive intestinal peptides and PACAP can inhibit IFN-γ-induced JAK1/STAT1 activation (42). Thus, we hypothesize that the activation of the JAK/STAT pathway may be involved in the pathogenesis of rosacea. Given the foundational role of the JAK/STAT signaling pathway in rosacea, JAK inhibitors hold promise as a prospective therapeutic paradigm for the management of rosacea. The possible mechanisms of JAK inhibitors in the treatment of rosacea may include inhibition of the inflammatory response, modulation of vascular permeability, inhibition of angiogenesis, and improvement of skin barrier function.

To date, three case reports have been documented, collectively enlisting a cohort comprising 25 individuals afflicted by rosacea, which have demonstrated benefit from JAK inhibition. Notably, these three case reports all report the utilization of tofacitinib in the therapeutic management of rosacea. Two of the case reports focused on 24 patients with refractory rosacea who used tofacitinib as monotherapy or adjunctive therapy and showed that tofacitib resulted in symptomatic improvement (43, 44). The remaining report showed the efficacy of tofacitinib in addressing steroid-induced rosacea (45).

Here, we present two cases of patients who received the JAK1 inhibitor upadacitinib and three cases of patients who received the JAK1 inhibitor abrocitinib for the treatment of refractory rosacea. Upadacitinib and abrocitinib may be promising medical options for patients with refractory rosacea. However, the long-term safety and efficacy of upadacitinib and abrocitinib require prospective controlled studies to assess them more comprehensively.

## Data availability statement

The raw data supporting the conclusions of this article will be made available by the authors, without undue reservation.

## Ethics statement

This study was approved by the Medical Ethics Committee of the West China Hospital of Sichuan University (approval number: 2024-237). Written informed consent was obtained from the individual(s) for the publication of any potentially identifiable images or data included in this article.

## Author contributions

TZ: Writing – review & editing, Writing – original draft, Validation, Project administration, Methodology, Investigation, Formal analysis, Data curation. XL: Writing – review & editing, Writing – original draft, Data curation, Conceptualization. LZ: Writing – review & editing, Resources, Methodology, Funding acquisition, Conceptualization. XJ: Writing – review & editing, Validation, Resources, Methodology, Conceptualization.

## Supplementary material

Supplementary Figure 1 Changes in psychological and quality of life and clinical manifestations and signs during abrocitinib treatment. **(A)** Case 5. **(B)** Case 6.
