# Supplementary figures and images for: Treatment of rosacea with upadacitinib and abrocitinib: case report and review of evidence for Janus kinase inhibition in rosacea
Source: Front Immunol. 2024 Jul 9;15:1416004. doi: 10.3389/fimmu.2024.1416004 (PMC11263021; doi:10.3389/fimmu.2024.1416004)

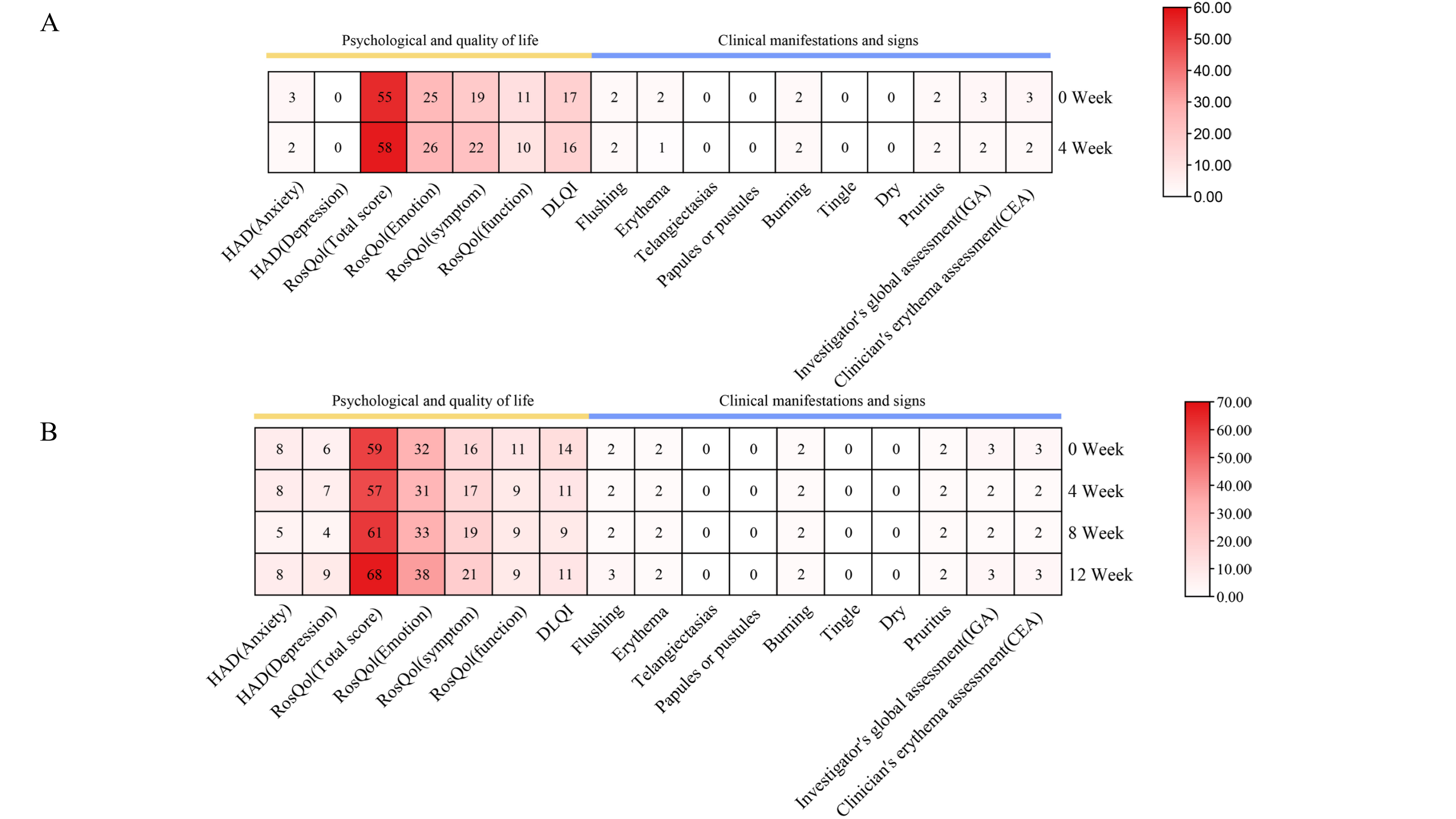

Supplement: Supplementary Figure 1 — Changes in psychological and quality of life and clinical manifestations and signs during abrocitinib treatment. (A) Case 5. (B) Case 6. [file Image_1.tif]
